# Advances and Future Prospects of Temperature and Salt-Resistant Gel Materials for Downhole Plugging Applications

**DOI:** 10.3390/gels11120955

**Published:** 2025-11-28

**Authors:** Junwei Fang, Peng Xue, Biao Wang, Jinsheng Sun, Yingrui Bai, Kaihe Lv, Yi Zhang

**Affiliations:** 1 School of Petroleum Engineering, China University of Petroleum (East China), Qingdao 266580, China; smart-byron@163.com (Y.B.); lkh54321@126.com (K.L.); 2 College of Chemistry and Chemical Engineering, Central South University, Changsha 410083, China; xuepeng416@126.com (P.X.); yzhangcsu@csu.edu.cn (Y.Z.); 3 College of Chemical Engineering, Xinjiang University, Urmuqi 830017, China; biaowang97@163.com

**Keywords:** downhole plugging, temperature and salt resistance, gel materials, bio-based degradable materials, nano-enhancement, self-healing

## Abstract

The exploitation of deep hydrocarbon resources in extreme environments, particularly high-temperature and high-salinity (HTHS) carbonate reservoirs, poses unprecedented challenges for downhole plugging operations. This review provides a critical analysis of the development of gel-based plugging materials designed to withstand these harsh conditions. It systematically examines three primary material categories—polymers, inorganic composites, and nanocomposites—dissecting the fundamental relationships between their molecular architectures and their resulting performance, including the pervasive trade-offs between mechanical strength, stability, and controllable degradation. While highlighting promising advances, such as bio-derived polymers and self-healing mechanisms, the review explicitly identifies the limitations of current technologies, most notably their inadequate long-term durability under synergistic HTHS stress and lack of industrial scalability. This forward-looking perspective emphasizes the integration of nano-reinforcements and stimuli-responsive chemistries as a critical pathway toward achieving the next generation of high-performance, deployable, and environmentally considerate plugging materials, thereby ensuring the efficient and sustainable development of challenging oil and gas assets.

## 1. Introduction

The ongoing global pursuit of hydrocarbon resources is driving the petroleum industry into increasingly complex geological and physicochemical environments, particularly deep and ultra-deep carbonate reservoirs. Characterized by high temperatures (>150 °C), high salinity (Total Dissolved Solids often exceeding 200,000 mg L^−1^), and intricate fracture-pore networks, these environments present a formidable challenge for wellbore stability and reservoir management. Under such harsh conditions, downhole plugging operations—essential for fluid loss control, zonal isolation, and enhanced recovery—are often hindered by polymer chain hydrolysis, syneresis, structural collapse, or uncontrolled degradation of conventional plugging materials, leading to operational failures and significant economic losses.

Confronting these limitations has spurred the development of advanced gel systems engineered for high-temperature and high-salinity (HTHS) resilience. Current research encompasses three major material classes: polymer gels, inorganic composites, and hybrid nanocomposites. While polymer gels offer chemical tunability and injectability, but generally suffer from a critical trade-off between mechanical strength and long-term stability. In contrast, inorganic composites provide robust structural integrity but lack the flexibility and degradability required for temporary plugging. Hybrid nanocomposites emerge as a promising avenue, leveraging nanoscale effects to synergistically enhance thermal stability, mechanical properties, and salt tolerance. However, the field remains fragmented, with most studies focusing on individual material improvements without a unifying framework that critically analyzes the fundamental performance trade-offs and pathways for integration.

This review moves beyond a descriptive catalog of materials to provide a critical analysis and a forward-looking perspective. It systematically deconstructs the design principles, performance boundaries, and failure mechanisms of state-of-the-art HTHS-resistant gel systems. By bridging molecular-level architecture with macroscopic plugging behavior, it seeks to clarify the fundamental factors governing gel performance and to outline a coherent research roadmap for the rational design of next-generation adaptive, multifunctional plugging agents.

## 2. Results and Discussion

### 2.1. Preparation and Performance Analysis of Polymer Materials

Gel-based plugging materials form a polymer network structure through crosslinking, exhibiting good elasticity and viscoelasticity, which makes them suitable for physical plugging and reservoir modification. However, under high-temperature and high-salinity conditions, the chemical stability of gels is readily compromised, showing issues like dehydration and degradation [1]. Resin-based plugging materials primarily rely on chemical reactions to form a cured plug in the formation, offering high plugging strength and stability. Nevertheless, overly rapid curing rates place stringent demands on construction conditions [2]. Cement-based plugging materials are characterized by high plugging strength and lower cost, but they struggle with uniform distribution in deep formations, often leading to formation bypass flow [3,4,5]. Polymer plugging materials form a tight plugging layer through controlled crosslinking reactions, demonstrating excellent plugging performance [6]. However, their stability under dynamic high-temperature environments still requires optimization [7]. Particulate plugging materials include flexible particles and delayed swelling particles. The former enhance plugging effects through chemical reactions, while the latter rely on water absorption and expansion to seal reservoir fractures [8]. Yet, both are prone to fragmentation under high-pressure formations, limiting their long-term application [9].

**Figure 1:**
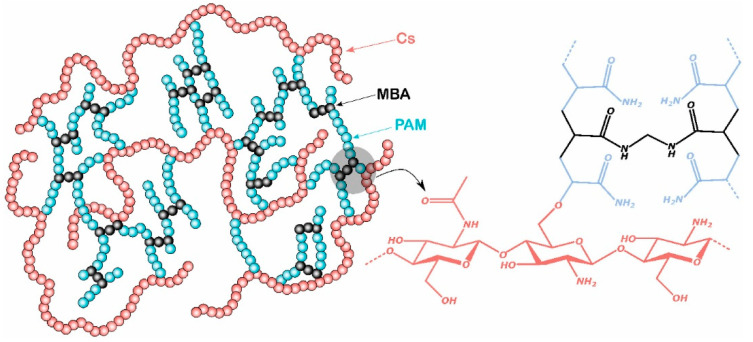
Crosslinking action between chitosan and polyacrylamide [11].

Among polymer materials, polyacrylamide-based polymers are widely used due to their outstanding plugging performance [10]. Through chemical modification and the introduction of crosslinking agents (such as chitosan-g-polyacrylamide, Figure 1), these materials can maintain stable performance under high-temperature (>120 °C) and high-salinity (>30% NaCl) conditions [11]. This research indicates that these materials can be combined with degradable characteristics, further optimizing reservoir protection and oil recovery efficiency.

Intelligent polymer materials, such as hydrogels and shape memory materials, are attracting significant attention due to their dynamic response characteristics [12,13]. Hydrogels are a type of polymer network formed by three-dimensional crosslinking, possessing high liquid absorption capacity and mechanical strength, making them suitable for plugging operations in high-permeability reservoirs. By optimizing molecular structures, the plugging performance of polymer materials can be enhanced [14,15]. For instance, introducing N-isopropylacrylamide (NIPAM) groups confers thermoresponsive properties to hydrogels, enabling adaptive plugging in downhole environments under high-temperature conditions [16]. Shape memory polymers undergo morphological changes triggered by specific environmental conditions (such as temperature and pressure), exhibiting excellent crack adaptation. In fractured formations, these materials can rapidly return to their preset shapes after wellbore pressure release, filling fractures and improving plugging efficiency.

**Figure 2:**
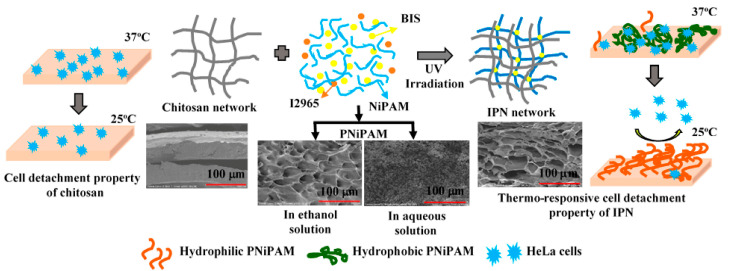
Crosslinking action of NIPAM [20].

Furthermore, the high resilience and stability of shape memory materials make them an ideal choice for future operations in complex reservoirs [17]. The degradability of polymer materials is another important property of concern for plugging applications. With increasing environmental protection demands in oilfields, the research and development of degradable plugging materials are receiving growing attention. These materials not only accomplish the plugging task but also reduce the long-term impact on reservoir production through controlled degradation. For example, combining polylactic acid (PLA) with PAM retains the high-strength plugging characteristics of PAM while achieving environmental friendliness through PLA degradation [18]. Additionally, by introducing ester bonds or hydrolyzable crosslinkers (Figure 2), polymer plugging materials can gradually decompose in specific downhole environments, avoiding long-term blockage of reservoir channels and enhancing subsequent development efficiency. After completing plugging or well repair operations, the degradation rate of these materials can be controlled by injecting an aqueous decomposition solution, further enhancing their practicality [19].

**Figure 3:**
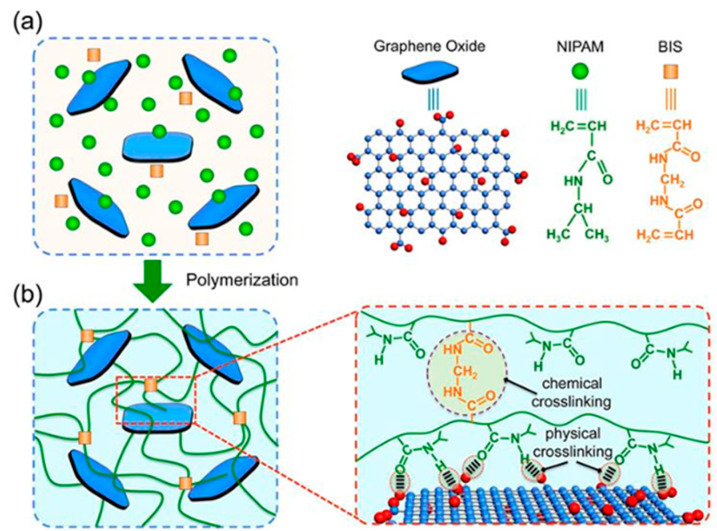
Schematic diagram of gel plugging a fracture [25]. (**a**) GO nanosheets are homogeneously dispersed in the monomer solution. (**b**) The PNIPAM-GO nanocomposite hydrogels are formed by both chemical and physical cross-linking, in which the PNIPAM chains are chemically cross-linked by BIS, and the hydrogen bond interactions between GO nanosheets and PNIPAM chains result in the physical cross-linking.

The primary polymer-based plugging materials are still gel-based. Gel-based plugging materials generate a dense network structure through the reaction between crosslinkers and polymers, forming a physical plug in the formation. This plug prevents water flow through dominant channels and forces fluid flow redirection, thereby enhancing water displacement efficiency. It also uses dynamic entrapment and adsorption [21,22,23]. Wang et al. [24] developed a physically crosslinked gel plugging agent (GP-A). Unlike the linear gel structure produced by HPAM, it is mainly composed of a spider-web-like star structure. The plugging mechanism is shown in Figure 3. This mechanism is a microcosm of the plugging mechanism of many gel-based plugging materials.

These plugging materials possess advantages such as good elasticity, high viscosity, strong toughness, and simple processing. However, they are prone to performance degradation in high-temperature and high-salinity formations due to chemical structure destruction and dehydration. Polymer hydrogel plugging materials alter the mobility ratio of oil and water through physical plugging and adsorption, achieving excellent profile control and plugging effects at low concentrations (typically 0.3–0.5%) [26]. Lei et al.’s [27] review pointed out that temperature-controlled crosslinked polymer plugging materials used in field of leak sealing operations exhibit high sensitivity to temperature changes, forming a stable plug in high-temperature environments and gradually restoring fluidity after cooling, making them suitable for short-term plugging operations in complex reservoirs. A practical example is Bai et al.’s [17] use of a polymer hydrogel based on acrylamide (PAM) and chromium salt crosslinkers. This hydrogel material is widely used in high-temperature and high-salinity reservoirs, capable of forming a tight plugging layer at temperatures up to 150 °C, achieving the desired plugging effect even at low concentrations. However, after gelation, polymer plugging materials gradually lose their fluidity. They are highly hydrophilic and have a relatively weak body structure. Their chemical structure is easily damaged in high-temperature and high-salinity reservoirs, making them unsuitable for operations requiring high strength [28,29].

Heavy oil-based plugging materials include coupled heavy oil, water-in-heavy oil emulsions, and activated heavy oil. These materials generate plugging capacity through emulsifiers that emulsify heavy oil into highly viscous water-in-oil emulsions [30]. Coupled heavy oil shows remarkable effectiveness in shale oil reservoirs and fractured formations, forming a stable plug under high-pressure conditions for profile control and enhanced oil recovery [31]. Yu et al. [32] prepared water-in-oil emulsions from heavy crude oil in the Xinjiang Oilfield, known as water-in-heavy oil. In field applications, this emulsion can form an effective plugging layer in porous media, improving fluid displacement efficiency in the reservoir. Experiments show that the oil phase in the emulsion can achieve stable plugging of fractured reservoirs through viscosity changes and interfacial tension adjustment. Activated heavy oil plugging materials combine surfactants to enhance emulsification efficiency, used for formation fracture repair and reservoir protection. Their adaptability to high salinity makes them an important tool for managing heterogeneous waterflooding reservoirs [33].

Resin-based plugging materials primarily consist of thermosetting resins such as urea-formaldehyde resin, epoxy resin, phenolic resin, and furfural resin. These materials undergo condensation reactions catalyzed by catalysts to form large macromolecules with a three-dimensional structure that are insoluble and infusible, effectively sealing fractures and pores [34,35]. Among resin-based plugging materials, thermosetting materials, represented by phenolic resin and epoxy resin, have found widespread application. For instance, phenolic resin-based plugging materials can form high-strength, insoluble solids at high temperatures (>200 °C) through catalyst-promoted condensation reactions, resulting in a significant sealing effect in deep fractured reservoirs [36]. Concurrently, epoxy resin-based plugging materials exhibit high application potential in high-pressure formations and complex reservoirs due to their excellent mechanical properties and chemical corrosion resistance, but their short curing time demands precise operational control [17]. However, the resin-based plugging materials have a relatively short reaction time, which causes them to gel before reaching the target plugging formation. Such premature gelling directly results in the materials failing to perform their plugging function. Additionally, resin-based plugging materials have the issue of short curing time, which requires the construction process to have extremely high precision control capabilities. Most conventional construction conditions cannot meet this requirement, thus limiting their application range [37,38]. These plugging materials each have their advantages and disadvantages, necessitating the selection of the most suitable type based on actual reservoir conditions and performance optimization for complex environments to enhance application effectiveness.

### 2.2. Structure Design and Application Research of Inorganic Composite Materials

**Figure 4:**
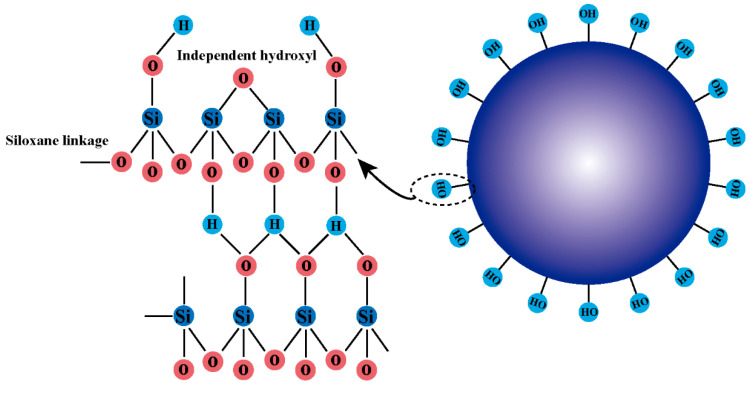
Silica Structure.

Inorganic composite materials utilize inorganic materials such as expanded graphite and nano-silica (Figure 4) as reinforcing matrices, enhancing plugging strength and high-temperature and salt-resistance capabilities through their structural characteristics [39,40]. For example, the incorporation of nano-alumina into cement-based materials significantly improves compressive strength and thermal stability under high-pressure conditions. Moreover, these materials possess good chemical inertness and environmental friendliness, making them suitable for deep-well operations [41].

**Figure 5:**
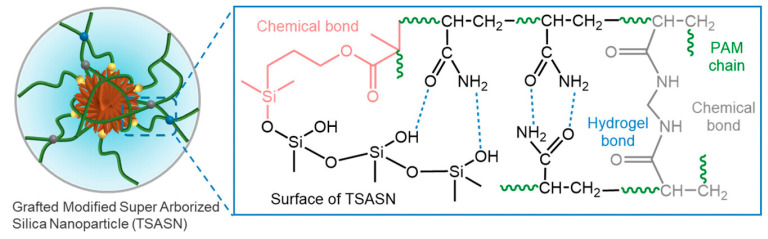
Silica Forming Hyperbranched Polymer [51].

Micro-silica (Silica fume) is a finely divided, high-surface-area silicon-based material often used as an additive in drilling fluids and plugging materials. Due to its uniform particle size distribution, micro-silica can fill fine fractures in fractured reservoirs, forming an impermeable seal [42,43]. Additionally, micro-silica exhibits excellent high-temperature resistance and chemical corrosion resistance, demonstrating superior stability under high-salinity conditions. Razzaq et al. showed that micro-silica, as a bridging agent in drilling fluids, can significantly reduce fluid loss and form a uniform sealing network due to its high surface area [42]. Yuan et al. further demonstrated that mixing micro-silica with cement matrices at a mass ratio of approximately 4:1 can improve the compressive strength of plugging materials and enhance their stability in high-temperature and high-salinity environments [5]. Furthermore, Al-Obaidi et al.’s review summarized that the application of micro-silica in water-based drilling fluids can reduce filtration loss rates, particularly exhibiting excellent micro-pore sealing effectiveness in fractured carbonate reservoirs [44]. Shu Zheng et al. [45], as well as others, have used modified nano-silica to create a new type of chemical gel plugging material. Pu et al. reported a novel water-soluble SiO_2_ core–shell hyperbranched polymer for EOR applications, with its 3D morphology conferring excellent shear resistance, salt tolerance, and thermal stability (Figure 5). Core flood experiments confirmed that the unique core–shell polymer may possess significant EOR potential [46].

Expanded graphite (EG) is an ideal material for high-temperature fracture sealing due to its high thermal expansion and unique layered structure. Its working principle involves rapid expansion at high temperatures, filling formation fractures and creating both physical and chemical sealing effects [47,48]. Sun Jingsheng et al. indicated that by compounding expanded graphite with a polymer matrix, the inadequate strength of graphite materials can be effectively compensated. Experiments showed that the composite material maintains stable expansion performance at 200 °C, achieving a sealing efficiency of over 95% [2]. Similarly, Zhao et al. studied the application of expanded graphite in high-salinity reservoirs, finding that its expansion coefficient in a saline environment is significantly superior to traditional particulate plugging materials [49]. Overall, inorganic composite materials often exhibit specialized capabilities in certain aspects compared to their raw material counterparts [50].

**Figure 6:**
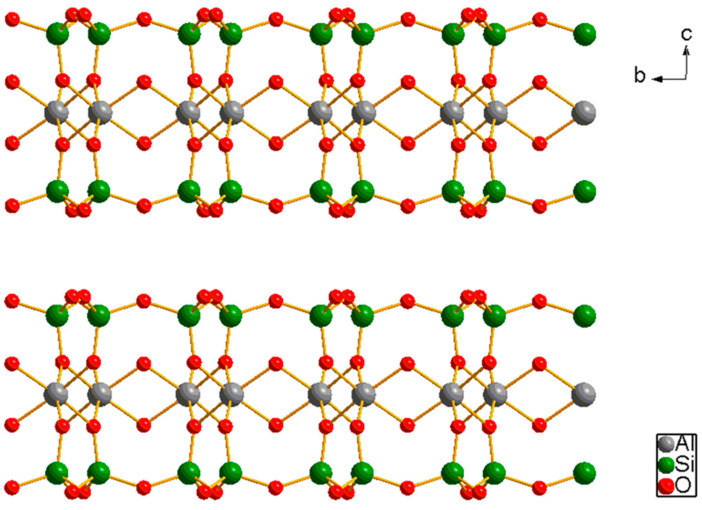
Structure of Sodium Bentonite [53].

**Figure 7:**
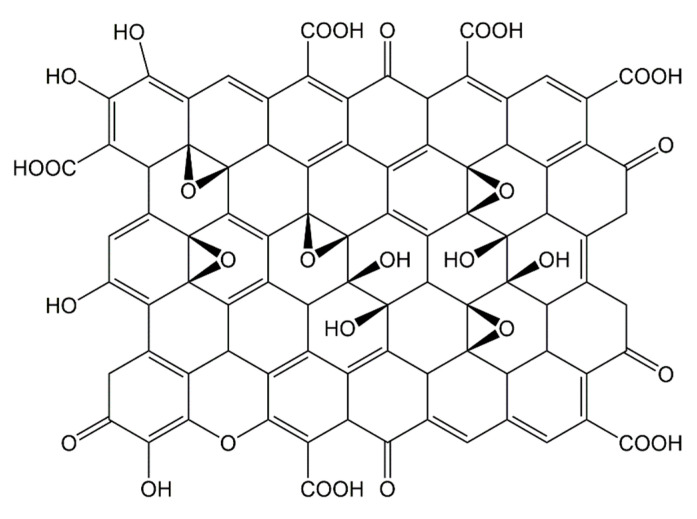
Structure of Graphene Oxide.

Hydrophilic swelling plugging materials are characterized by their ability to absorb water, expand in volume, and possess a certain degree of viscoelasticity and strength. Once introduced into the formation, they can plug formation pores, making them commonly used as plugging agents for profile modification in oilfields. Lin Shujie used an ammonium persulfate–sodium bisulfite redox system as an initiator, incorporating inorganic materials such as nano-silica, talcum powder, sodium bentonite (Figure 6), and graphene oxide (Figure 7), and employed solution polymerization to synthesize a novel high-temperature and salt-resistant hydrophilic swelling temporary plugging material [52].

Hao Chen developed a high-temperature resistant, curable elastic co-polymer gel temporary plugging material system with a temperature resistance of 160 °C, a stable period of more than 10 days, and a gelation time of 1–10 h [54]. The addition of sodium montmorillonite to the elastic co-polymer gel can significantly enhance the thermal stability of the co-polymer gel. The abundant hydroxyl groups on sodium montmorillonite form hydrogen bonds with water molecules, increasing the amount of bound water and further improving the high-temperature resistance of the co-polymer gel.

Meng Liyan et al. investigated the influence of inorganic material (calcium bentonite, sodium carbonate, etc.) content and crosslinker usage on product performance. The results showed that the addition of an appropriate amount of inorganic materials and the use of crosslinkers can significantly improve the comprehensive properties of the polymer. Wang Jianjun [55] studied the effects of incorporating fibrous, flaky, and particulate inorganic materials into gel systems, finding that they can increase the strength and viscosity of the gel after gelation. Moreover, calcium carbonate particulate materials and sepiolite fiber materials were found to enhance the P(AM-co-AA) polymer gel, significantly increasing the system’s strength and shortening its gelation time. Yuan Zhe et al. [56] also studied foam flooding, an effective technique in the thermal recovery of heavy oil reservoirs. They designed and conducted a series of nitrogen foam steam flooding sandpack tests, comparing influencing factors such as gas–liquid (foaming agent) ratio, permeability, injection scheme, and oil saturation to evaluate foam stability and blockage mechanisms. It was found that foam selectively blocks larger pores in porous media with high permeability, a characteristic beneficial for enhancing the sweep efficiency and oil recovery in heavy oil reservoirs. Tang Xiaofen et al. [57] developed a non-toxic and environmentally friendly inorganic gel coating diverter, specifically designed for deep water injection to enhance water flooding effectiveness. After injection into the formation, the agent reacts with formation water to form an inorganic silicate gel with a density close to that of formation water. The gel, in bulk or particulate form, suspends in water, adheres to the rock surface through adsorption, and forms an inorganic gel coating. This narrows the preferential flow paths of deep water, creating flow resistance, forcing subsequent fluids to diverge, thereby improving water flooding effectiveness. This agent is characterized by rapid dissolution, high-temperature resistance, high-salinity tolerance, environmental friendliness, and long-lasting effectiveness. It has been applied for deep flow profile control in various oil reservoirs with different temperature and salinity conditions, such as the Lunnan oilfield in the Tarim Basin, the Yuejin oil area in the Qaidam Basin, and the Dagang oilfield.

### 2.3. Synthesis Methods and Property Characterization of Nano-Materials

Nano-materials often appear in combination with the aforementioned categories [58,59,60]. Due to their unique size effects and excellent physicochemical properties, they have demonstrated significant potential in multifunctional plugging materials for high-temperature and high-salinity environments in recent years [61,62]. Additionally, the rich modifying of nano-materials lays the foundation for the development of many new materials [63,64,65].

**Figure 8:**
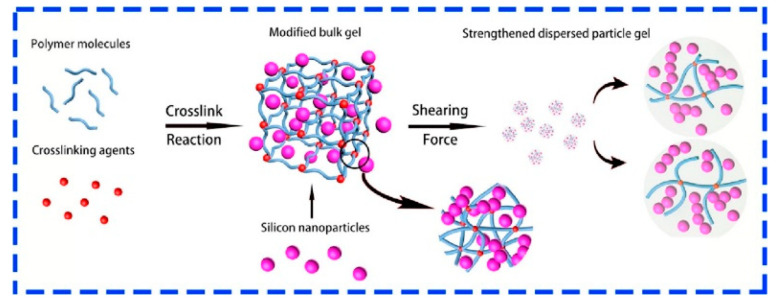
Mechanism of Silica Nanoparticle-Stabilized Gel Plugging Material [66].

Research has shown that the composite design of polymers and nano-silica particles can achieve higher plugging strength and faster degradation rates (Figure 8) [66,67]. The high specific surface area and adjustable surface chemistry of nano-materials enable them to possess outstanding salt resistance in high-salinity environments. By introducing functional groups (such as carboxyl and sulfonate groups) onto the surface of nanoparticles, the adverse effects of salt ions can be effectively mitigated, preventing the deterioration of material properties [68,69]. Xu et al. investigated the application of wet-phase modified expanded graphite particles in high-salinity carbonate reservoirs, where the nano-scale dispersion significantly enhanced the plugging performance of the particles [70]. Furthermore, Liu et al. employed nano-modification techniques to improve the adaptability of traditional plugging materials in high-salinity reservoirs, exhibiting excellent salt resistance stability, particularly in porous media [71]. The composite use of nano-materials can significantly enhance stability under high-temperature and high-salinity conditions, but further optimization of degradation performance is often required [72,73].

Biodegradable plugging materials, serving as temporary blocking agents, have demonstrated superior performance and application prospects in high-temperature and high-salinity reservoirs [74,75]. Through the optimization of formulation and environmental parameters, the application of biodegradable materials in multi-stage stimulation has been significantly improved, particularly in terms of stability and degradation characteristics under high-temperature conditions [76]. Tu indicated that such materials can effectively achieve temporary plugging in oil and gas wells [77]. Polymer-based biodegradable drilling fluid systems have been successfully applied in coalbed methane wells, and the compatibility of these materials under high-temperature and high-salinity conditions has verified their unique advantages over traditional plugging materials, such as excellent plugging capacity and stable mechanical properties [78]. In the research of enhanced oil recovery (EOR) techniques, the high-temperature and high-salinity resistance of polymer-based plugging materials has been further optimized, along with good shear resistance, improving the efficiency of complex reservoir development [79]. In addition, research on intelligent polymer plugging materials has shown that molecular structure adjustment can enhance their toughness and environmental adaptability, although further optimization is needed to overcome performance shortcomings in high-pressure environments [80]. In the development of new materials, preformed particle gels (DPPG), as an innovative biodegradable material, exhibit good plugging capacity and controllable degradability in high-temperature environments through the optimization of raw material ratios, providing technical support for the efficient development of oil and gas fields [81]. The study of temperature-controlled degradable plugging materials has further expanded the application range of degradable materials in complex reservoirs, achieving precise material response through temperature-sensitive mechanisms and successfully addressing the challenges of high-temperature reservoirs [82,83]. Furthermore, acid-sensitive biodegradable gel plugging materials have been developed for acidic fracturing operations, with their environmentally friendly characteristics and efficient plugging performance being particularly suitable for operations with high environmental requirements [84]. Environmentally friendly biodegradable drilling fluids have also been validated in reservoir development; especially when combined with microbial degradation technology, these materials exhibit significant application potential [85,86,87]. In specific applications, Guo et al. developed a high-temperature biodegradable polymer plugging agent that demonstrated excellent network structure and efficient plugging performance in nuclear magnetic resonance tests, further consolidating the technical foundation in this field [88]. Ye et al. proposed a self-degradable plugging agent, SDPF, which exhibits self-degradation ability under high-pressure and acidic conditions, having significant importance in protecting reservoirs and sealing fractures [89]. Qiao et al.’s starch-based temporary plugging material stands out in terms of salt resistance, high-temperature tolerance, and environmental compatibility, providing a low-cost and highly efficient solution for temporary plugging operations in complex reservoirs [76]. Through the introduction of intelligent response mechanisms and improvements in molecular design, greater breakthroughs are anticipated in this field in the future.

However, the research, development, and large-scale application of biodegradable plugging materials still face many challenges. For example, balancing high-temperature and salt resistance with high strength and degradation characteristics is a key issue. High-temperature and salt-resistant plugging materials need to maintain chemical and mechanical stability under extreme conditions. However, there is a significant contradiction between the thermal stability and degradation characteristics of existing materials [90]. For instance, nano-composite plugging materials improve high-temperature tolerance through the incorporation of silicates and polymers, but their degradation ability significantly declines after prolonged exposure in the reservoir [91]. Sun’s research indicates that in fractured reservoirs, plugging materials must simultaneously satisfy high compressive strength and dynamic response capabilities. However, high-strength plugging materials typically exhibit poor degradation performance at low temperatures, necessitating further optimization of their molecular structure [62]. In addition, some high-performance materials (such as sulfonate-modified polymers) are costly, limiting their large-scale industrial application. In production, there are constraints related to the need for low-cost and efficient production technologies. High-performance plugging materials rely on specialty monomers and additives, particularly sulfonate-modified polymers and nano-particles, which are expensive [92]. The difficulty in balancing plugging and degradation properties under high-temperature and high-salinity conditions is highlighted by the geothermal well project that utilized polylactic acid (PLA)-based plugging materials. The material was designed to improve reservoir permeability through degradation and pore release. However, due to the excessively rapid degradation rate, the integrity of the reservoir plugging layer was compromised [93].

Currently, plugging materials are applied in a site-specific manner in many oilfields [94,95,96]. Ma et al. comprehensively analyzed the research progress of nano-plugging materials and their practical application in drilling fluids [97] ce area and excellent particle size distribution, can effectively fill micro-fractures, enhancing formation stability. In a fractured reservoir in the southwestern region, the use of nano-plugging materials successfully reduced fluid loss by 20%, while also improving the stability of the drilling fluid, significantly lowering drilling costs. Chen et al. verified through field experiments that modified nano-plugging materials can rapidly form a tight plugging layer under high-pressure conditions, effectively preventing fluid loss. After the application of this technology in an eastern oilfield, the plugging efficiency was increased to over 90%, and the service life of the wellbore was significantly extended [98]. Liu et al. demonstrated the application potential of these materials in high-salinity reservoirs through specific case studies. In a high-salinity reservoir, the silicate chemical water shut-off technology effectively addressed severe fluid loss issues by adjusting the concentration of the plugging material and the gelation time. Field data showed a reduction in fluid loss of 80%, with a marked improvement in reservoir protection [99]. Han et al. employed a composite plugging slurry consisting of high-temperature rigid materials (DXD) and flexible graphite materials (TXD) in high-temperature and high-pressure wells in the Yinggehai-Qiongdongnan Basin, addressing well leakage issues through a combination of bridging and fracture filling. This technology has been applied in several high-temperature and high-pressure wells, increasing the success rate of plugging from the traditional 30% to 80%, providing an efficient solution for well leakage management in complex reservoirs [100].

### 2.4. Key Properties Comparison of Plugging Materials

To systematically evaluate and compare the applicability of different types of plugging materials, the table below summarizes the typical parameters of key physical and physicochemical properties for polymer materials, inorganic composites, and nanocomposites, among others (Table 1 and Table 2). These parameters, including shrinkage coefficient, heat capacity, density, and mechanical strength, are crucial for predicting material performance under high-temperature.

In summary, polymer-based materials exhibit excellent injectivity and degradability but are limited by insufficient mechanical strength and long-term thermal stability. In contrast, inorganic composites possess outstanding temperature resistance and structural robustness, yet they generally lack controllable degradation and removability. Nanocomposites, by integrating the advantages of both systems, offer a more balanced combination of properties and represent a promising direction for next-generation high-performance plugging materials. It becomes possible to synergistically optimize plugging strength, durability, and controllable degradability through rational molecular-level design, thus establishing a solid technological foundation for efficient and sustainable hydrocarbon development under extreme downhole conditions.

## 3. Conclusions

This review summarizes the current progress of temperature and salt-resistant gel materials for downhole plugging. Conventional AM/AMPS-based systems have established a performance benchmark, typically tolerating conditions of up to 140 °C and 200,000 mg L^−1^ total dissolved solids. However, its development remains limited by inherent performance antagonisms, particularly between mechanical robustness and degradability, as well as between salt tolerance and injectivity or economic efficiency under extreme conditions. Future development relies on molecular design strategies that balance strength and adaptability under harsh conditions. Dynamic and delayed crosslinking mechanisms, addressing the core challenge of crosslinking kinetics in HTHS environments, together with structural reinforcement through fluorination, steric protection, or nanoscale fillers, show great promise for improving both stability and durability. Recent advances in hybrid and nanocomposite gels have demonstrated synergistic improvements in mechanical integrity and salt tolerance, while biomimetic and stimuli-responsive materials offer new directions for selective and autonomous plugging in complex reservoirs. Overall, the transition from single-component gels to adaptive hybrid systems marks a key step toward cost-effective, high-performance plugging materials. The strategies outlined here provide valuable guidance for developing the next generation of intelligent plugging gels for harsh oilfield environments.

## Figures and Tables

**Table 1:** Comparison of Physical and Physicochemical Properties of Polymer Materials, Inorganic Composites, and Nanocomposites.

Property	Polymer Materials [101,102,103,104,105]	Inorganic Composite Materials [106,107,108]	Nanocomposite Materials [109,110,111,112,113,114,115,116,117,118,119,120]
Density (g/cm^3^)	1.0–1.2	1.5–2.2	1.1–1.8
Mechanical Strength	Elastic Modulus: 10^2^–10^4^ Pa Compressive Strength: 10–60 MPa	Compressive Strength: 20–80 MPa	Compressive Strength: 30–100 MPa Storage Modulus (G’): 10^2^–10^5^ Pa
Shrinkage/Coefficient of Expansion	~70–200 × 10^−6^/K	~5–15 × 10^−6^/K	~20–100 × 10^−6^/K
Heat Capacity (J/g·K)	~1.5–2.5	~0.8–1.2	~1.0–2.0
Max. Service Temperature (°C)	120–200	200–400+	150–300+
Salinity Tolerance (TDS)	Moderate to High	Very High	Very High
Degradability/Removability	Good to Excellent	Poor	Good

**Table 2:** Comparison of gel materials and non-gel materials (resins and cements) highlighting their advantages and disadvantages.

Material Type	Advantages	Disadvantages
Gel Materials	Tunable properties, Self-healing, Enhanced by hybrid systems, multifunctional [121]	Limited robustness, Sensitive to stress
Non-Gel Materials	Strong, durable, stable for structure [122]	Not self-healing, lacks adaptability, Limited biocompatibility
(Resins, Cements)	Good for long-term sealing [123]	Fewer functions, not responsive

## Data Availability

No new data were created or analyzed in this study.
